# Collaborative Management of Opioid Use Disorder and Cancer Care in a Patient With Medical Complexity and Housing Instability

**DOI:** 10.7759/cureus.105337

**Published:** 2026-03-16

**Authors:** Elizabeth A Abrams, Margaret Williams

**Affiliations:** 1 Internal Medicine, Addiction Medicine, The Ohio State University Wexner Medical Center, Columbus, USA

**Keywords:** cancer and non-cancer pain, inpatient care coordination, medication for opioid use disorder, opioid use disorder, unstable housing

## Abstract

A 41-year-old female with a past medical history of depression, anxiety, intravenous drug use, and opioid use disorder (OUD) presented to the emergency department in December with frostbite of her bilateral hands and feet after living outside. She was found to have a left brachial artery occlusion and underwent embolectomy the next day. Her 51-day hospital course was subsequently complicated by an ST-elevation myocardial infarction, heart failure with reduced ejection fraction, stroke, right brachial vein deep vein thrombosis, pyelonephritis, acute hypoxic respiratory failure, and a new diagnosis of stage IV vulvar and anorectal squamous cell carcinoma, necessitating ostomy placement. Her frostbite also led to significant debility of her legs.

The patient had a 20-year history of using opioids, and at the time of index admission, smoked 1 gm of fentanyl daily. Throughout her hospital course, the Addiction Medicine, Palliative Care, Psychiatry, Gynecologic Oncology, Cardiology, and General Medicine teams collaborated to manage her pain, cravings, and withdrawal, and to coordinate care in the setting of multiple comorbidities. For OUD and pain, she was initially started on methadone, then switched to buprenorphine due to prolonged QTc, and discharged when stable. However, she relapsed on fentanyl during her time out of the hospital, and upon readmission was ultimately switched back to methadone through close coordination with all teams, including Cardiology. The patient was stabilized on a regimen of methadone, gabapentin, and duloxetine, with several other medications tried and provided as needed for pain management throughout her admissions.

Her medical teams also advocated to the hospital leadership to allow her to remain inpatient for her radiation therapy, given her insecure housing situation; she ultimately completed all radiation and chemotherapy treatments. Particularly given that guidance for the management of OUD in patients with advanced cancer is relatively nascent, this case demonstrates innovative, multidisciplinary management of a patient with significant medical and psychiatric complexity, multiple pain syndromes, and successful completion of inpatient cancer treatment in a patient with OUD and housing insecurity.

## Introduction

The trimorbidity of physical illness, mental illness, and substance use disorder disproportionately affects people experiencing homelessness and results in excess mortality relative to housed individuals [1,2]. The presence of trimorbidity in a person experiencing homelessness has been associated with over four times the odds of unplanned hospital admission compared to those experiencing homelessness without trimorbidity [3]. These circumstances are often more difficult for unsheltered homeless individuals, as those who are unsheltered have higher levels of health risk than sheltered homeless populations [4]. While multifactorial, drivers of these disparities include challenges in obtaining and sustaining engagement with coordinated physical, mental health, and substance use disorder care.

We present a case of a 41-year-old unhoused female who initially presented with frostbite, leading to a medically complex admission, including a new diagnosis of stage IV vulvar squamous cell carcinoma, ST-elevation myocardial infarction (STEMI), and severe ischemic cardiomyopathy. At the time of admission, she had been living outdoors in a tent. She also had depression, anxiety, and opioid use disorder (OUD), necessitating close collaboration between the Addiction Medicine, Palliative Care, Psychiatry, Gynecologic Oncology, Cardiology, and General Medicine teams to manage her pain, cravings, and withdrawal and coordinate care in the setting of multiple comorbidities. We aim to inform clinical practice by highlighting strategies for comprehensive, quality patient care and chronic disease management that account for the complexity of social and clinical conditions.

## Case presentation

A 41-year-old female with a past medical history of intravenous drug use and OUD presented to the emergency department in December with frostbite of her bilateral hands and feet after living outside. She was found to have a left brachial artery occlusion and underwent embolectomy the following day. Her 51-day hospital course was subsequently complicated by a STEMI, heart failure with reduced ejection fraction (HFrEF), stroke, right brachial vein deep vein thrombosis, pyelonephritis, acute hypoxic respiratory failure, and a new diagnosis of stage IV vulvar and anorectal squamous cell carcinoma, necessitating ostomy placement. Her frostbite led to significant debility of her legs, requiring maximum assistance from sit to stand seven weeks into index admission. She was subsequently readmitted multiple times with chest pain, influenza, and pulmonary embolisms. Each complication introduced a layer of medical complexity to the case, and took place in the setting of homelessness, OUD, and loss of her husband to endocarditis the day prior to index admission.

The patient had a 20-year history of using opioids, and at the time of admission, smoked 1 g of fentanyl daily. She had been to inpatient withdrawal management and residential treatment programs and had tried both buprenorphine/naloxone and methadone many years prior. She reported that “having a good support team” helped her not use opioids during periods of sobriety. She also occasionally used crack cocaine and formerly smoked cigarettes, but quit 11 years prior.

Throughout admission, the Addiction Medicine, Palliative, and Psychiatry teams collaborated to treat her OUD, depression, anxiety, and pain. She was initially on as-needed hydromorphone and oxycodone after her embolectomy. After several days of being hesitant to start buprenorphine due to concern about precipitated withdrawal, the patient started methadone at 10 mg. However, her QTc was high (563), so the decision was made to rotate to buprenorphine due to cardiac concerns, especially in the setting of her HFrEF and recent STEMI. She successfully completed a cross-taper from methadone to buprenorphine (Belbuca), followed by buprenorphine/naloxone, and was discharged with maintenance buprenorphine/naloxone. Palliative and Addiction Medicine teams worked with her to reduce her pain regimen to include only an as-needed, oral, full agonist opioid to optimize discharge planning, so they began tapering down and ultimately discontinued oxycodone and continued oral hydromorphone. 

After her index stay, the patient was discharged and readmitted to the hospital soon thereafter for a total of nine times in the next six months. She was readmitted one day later twice, within one week of discharge five times, and readmitted within two weeks of discharge twice. These readmissions were most often for chest pain and recurrent pulmonary embolisms. After her third admission for chest and abdominal pain, the patient reported relapse on fentanyl while prescribed buprenorphine/naloxone outpatient and noted uncontrolled pain while on buprenorphine/naloxone. She used 0.5 g of fentanyl daily outside the hospital, a reduction from her pre-index admission dose of 1g per day. Given the patient’s ongoing fentanyl use, preference for methadone over buprenorphine, and methadone’s analgesic potential [5], the team discussed with Cardiology whether a switch back to methadone would be appropriate. Cardiology re-evaluated the patient’s QTc using the Bazett formula, given her right bundle branch block, and found it to be lower at 459. Cardiology agreed that treating the patient’s OUD was essential to her recovery from HFrEF and malignancy, and therefore supported the decision to restart methadone. All parties observed and recognized that the best form of medication for OUD was the form the patient would be invested in taking, and that abstinence from illicit opioids would promote adherence to other recommended medical treatment plans. She started methadone with subsequent dose titration to 80 mg at discharge. Upon later readmissions, this dose was adjusted to 30 mg.

**Figure 1:**
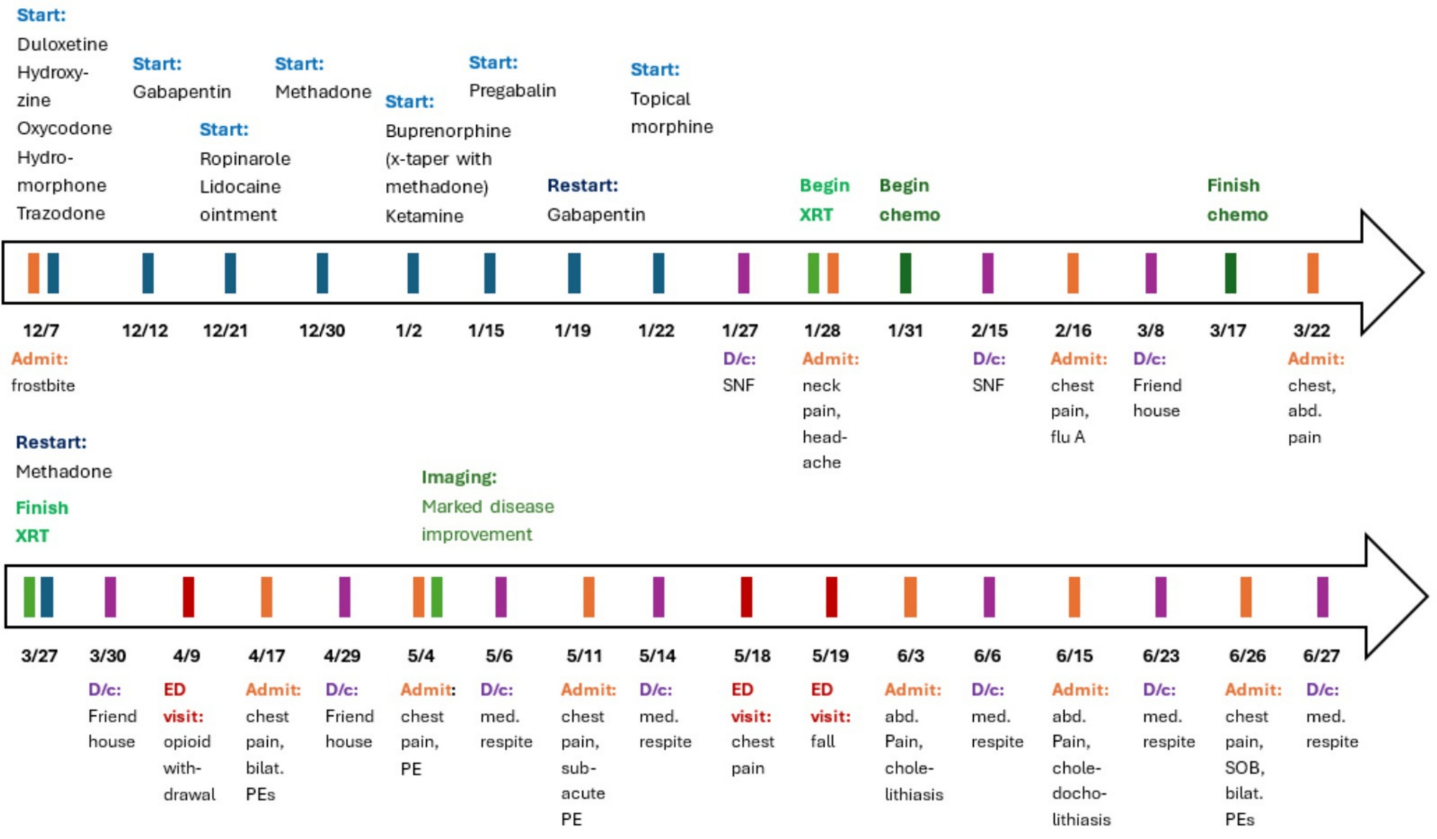
Milestones of the treatment course and medication management This figure demonstrates the patient’s care and treatment over the course of six months. Her multiple readmissions underscore the improbability of her being able to survive outside the hospital while facing significant medical complexity and systemic social barriers. Therefore, completing the majority of her cancer care while inpatient was a significant asset to the successful treatment of her malignancy. Abd. = Abdominal; Chemo. = Chemotherapy; D/c = Discharge; Med. Respite = Medical Respite; PE = Pulmonary Embolism; SNF = Skilled Nursing Facility; SOB = Shortness of Breath; XRT = External Beam Radiation Therapy; X-taper = Cross-taper

The patient was also started on duloxetine during her first admission for depression and anxiety, as well as neuropathic pain from her frostbite. On recommendation from Psychiatry, this was uptitrated to a dose of 90 mg daily. She also took gabapentin, with the dose increased to 800 mg TID. Ropinarole, lidocaine ointment, and topical morphine were added to address restlessness and ongoing pain in her lower extremities secondary to frostbite. Ketamine and pregabalin were also temporarily tried while inpatient, and as-needed trazodone and hydroxyzine were also available. A timeline of the patient’s MOUD and pain management plan is included in Figure 1.

She was seen multiple times by Certified Peer Recovery Specialists throughout every admission, who helped her understand and think about various treatment decisions. The Peers validated her concerns, helped her navigate grief related to her husband’s recent death and lack of contact with other family, provided support when initiating buprenorphine and methadone, and listened when the patient expressed fear and distress about her many new diagnoses.

**Figure 2:**
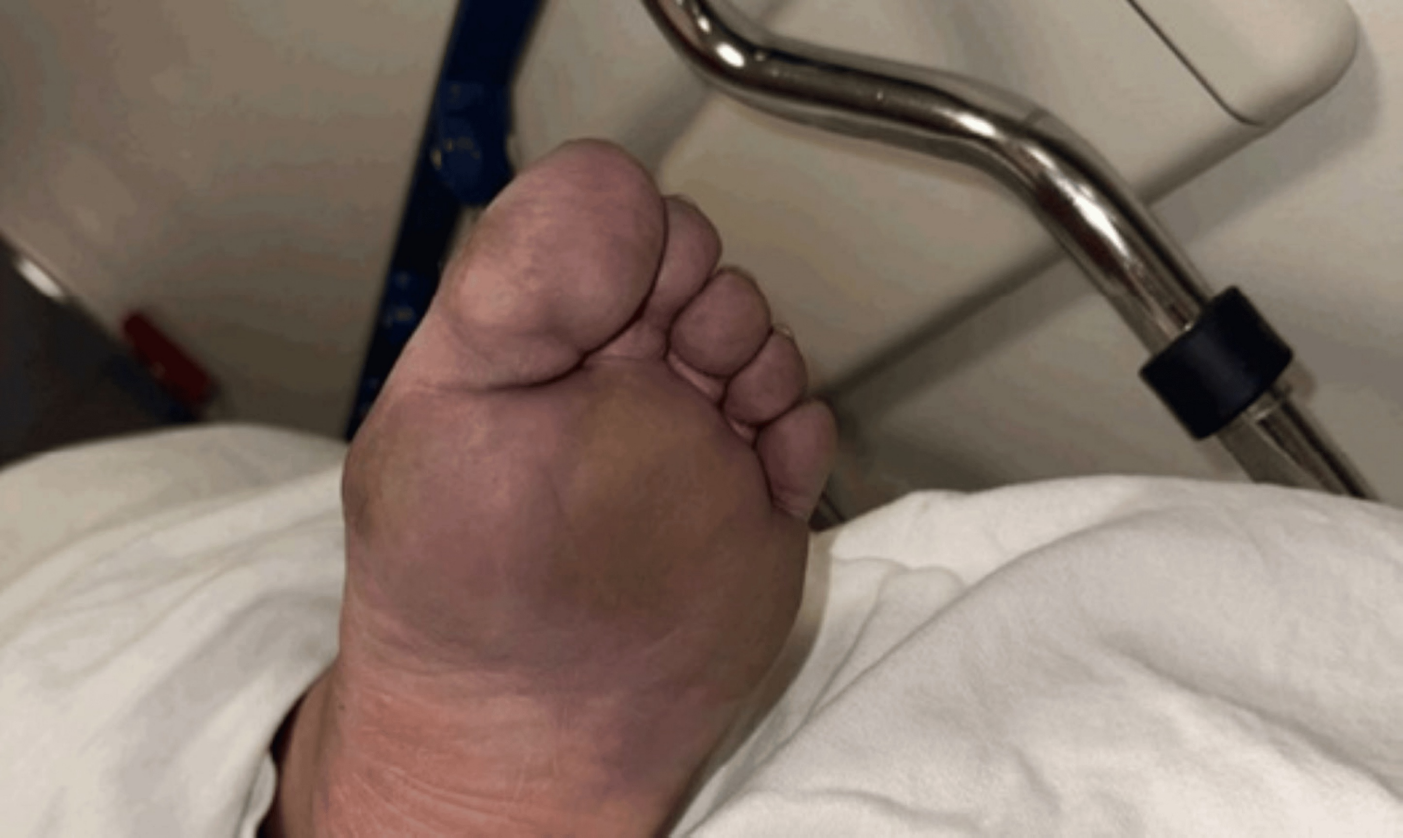
Frostbite at the index admission The patient’s severe frostbite and associated pain and neuropathy significantly limited her mobility and posed barriers to accessing treatment for cancer, opioid use disorder, and other co-morbidities outside the hospital.

**Figure 3:**
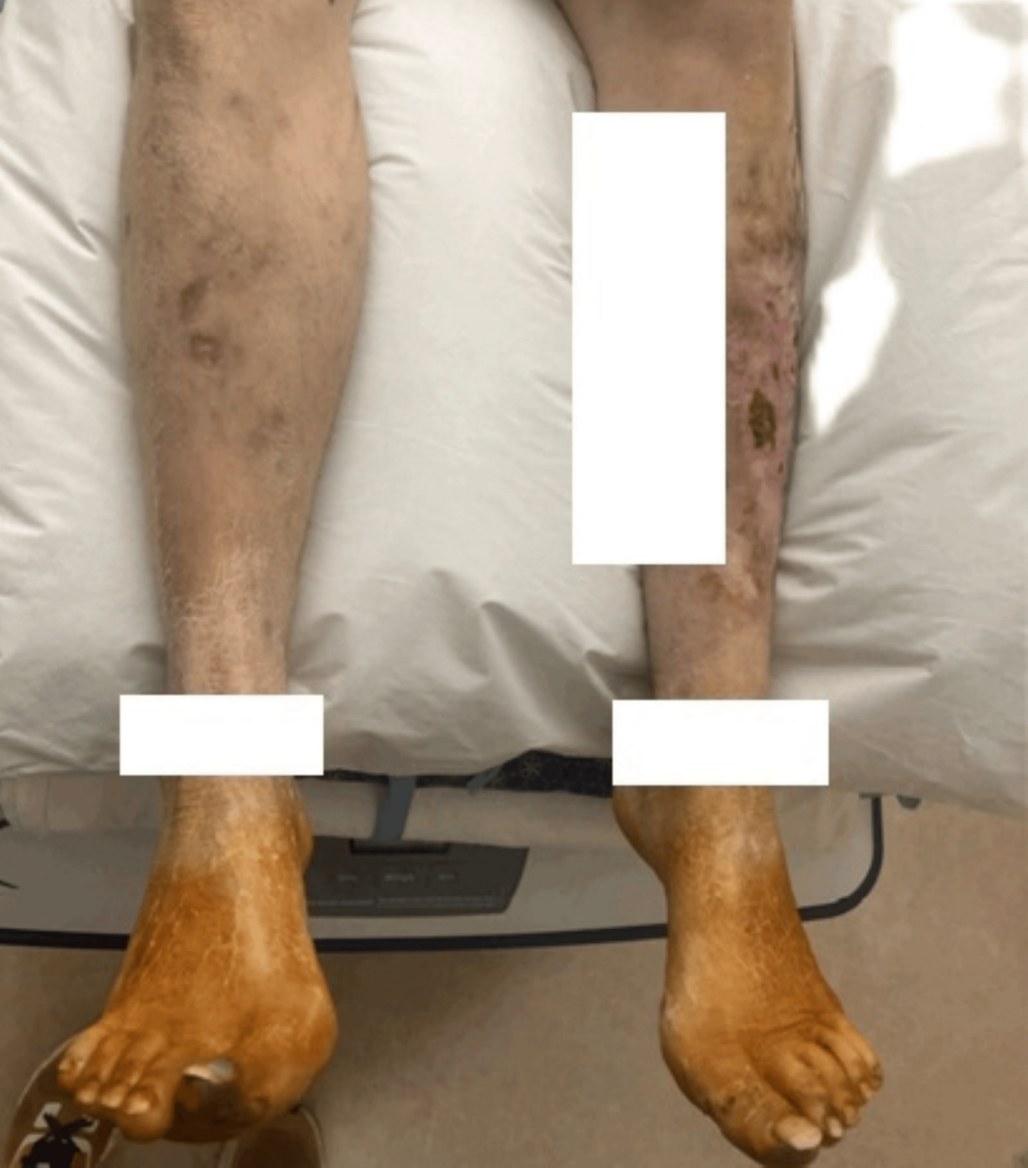
Lower extremities 52 days after the index admission on the patient's first day of radiation therapy The patient’s severe frostbite and associated pain and neuropathy significantly limited her mobility and posed barriers to accessing treatment for cancer, opioid use disorder, and other co-morbidities outside the hospital.

Additionally, her Addiction Medicine and Palliative teams advocated strongly to the hospital leadership that the patient be allowed to stay in the hospital to begin her radiation treatments for her newly diagnosed vulvar cancer. She was initially going to be discharged to a Long-Term Acute Care (LTAC) Facility, but insurance coverage did not allow for her to leave the LTAC for outside radiation appointments. She also had significant mobility limitations due to her frostbite and neuropathy. Figures 2-3 show her lower extremities on day 2 of admission and one month later.

Table 1 shows the patient's Activity Measure for Post-Acute Care (AMPAC) and pain scores as assessed by periodic physical and occupational therapy assessments throughout hospitalization. AMPAC scores assess functional status and mobility, predicting risk of discharge to a post-acute care facility [6]; the patient’s scores met criteria for high risk for the first three months after index admission, and moderate risk for the following three to four months. She also remained with severe pain for over three months after the initial admission.

**Table 1:** Activity Measure for Post-Acute Care (AMPAC) and pain scores throughout hospitalization Assessments of the patient’s AMPAC and pain scores throughout hospitalization suggest significant mobility and pain limitations affecting her ability to access care outside the hospital. *AMPAC is a validated Basic Mobility score assessed by Physical and Occupational Therapists and used to predict discharge destination. Scores range from 6 to 24, with higher scores indicating better mobility. Scores are stratified into three categories of risk for requiring post-acute care facility at discharge: <15 = high-risk, 15-19 = moderate risk, >19 = low risk. ^Pain was assessed on a scale from 1-10 by Physical and Occupational Therapists, with higher scores indicating more severe pain.

	AMPAC*	Pain at Rest^	Pain With Activity^
8-Dec	13	10	10
17-Dec	10	10	10
27-Dec	9	9	10
10-Jan	13	10	10
21-Jan	14	8	8
23-Jan	14	7	8
30-Jan	13	7	8
5-Feb	11	0	Not documented
19-Feb	18	8	10
21-Apr	17	0	0
25-Apr	19	0	0
28-Apr	18	9	9
19-Jun	21	Not documented	Not documented
20-Jun	18	Not documented	Not documented
23-Jun	19	Not documented	Not documented

**Figure 4:**
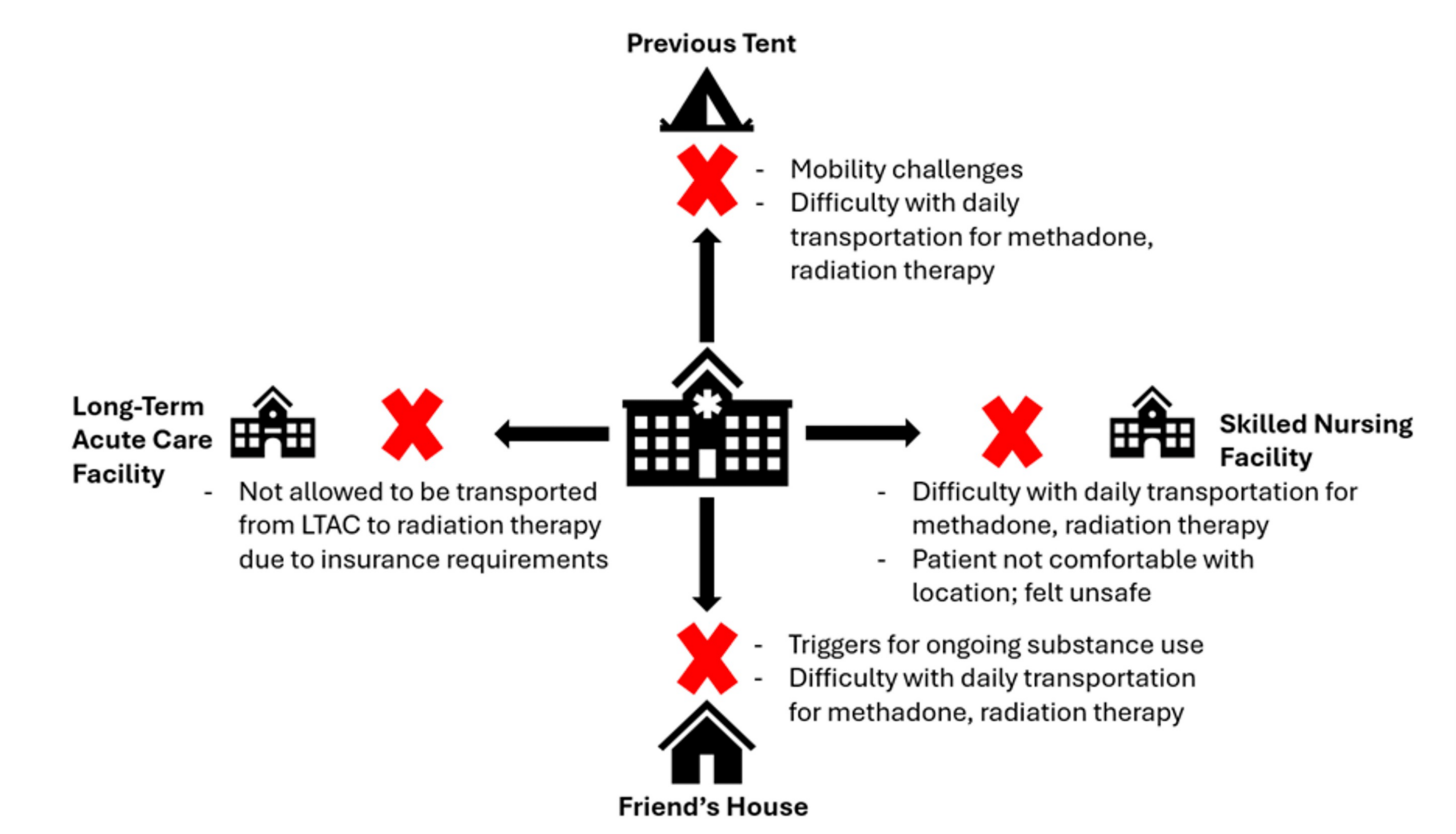
Barriers associated with possible discharge locations The multidisciplinary team considered several discharge locations for this patient, including a skilled nursing facility, long-term acute care facility, and her friend’s house. Systems-level barriers (e.g., insurance, transportation) and individual-level concerns meant none of these were appropriate options for the patient’s success and ongoing health maintenance outside the hospital. LTAC: Long-Term Acute Care Image credit: The authors, using Microsoft PowerPoint (Microsoft Corporation, Redmond, WA, US)

Together, these factors compounded the difficulty of returning to the hospital for radiation treatments or getting to a methadone clinic daily. Figure 4 shows the barriers associated with the patient’s possible discharge locations.

This meant that there was no optimal discharge option that would allow her to continue treatment for cancer, OUD, and physical therapy. Given these multifactorial challenges, permission was given to her Primary and Gynecologic Oncology teams for the patient to remain hospitalized to complete radiation and chemotherapy successfully.

Table 2 summarizes key features of the case.

**Table 2:** Key features of the case

Key Feature	Case Details
Demographics	41-year-old female.
Medical History	Opioid use disorder, intravenous drug use.
Social History	Lived outside. Husband passed away one day prior to the index admission. Used opioids for 20 years. Occasional crack cocaine use. Stopped smoking cigarettes 11 years prior to admission. Limited social support.
Presenting symptoms	Frostbite of the bilateral hands and feet during winter.
Complications	New diagnosis of stage IV squamous cell carcinoma, left brachial artery occlusion, ST-elevation myocardial infarction, heart failure with reduced ejection fraction, stroke, deep vein thrombosis, multiple pulmonary embolisms, pyelonephritis, and acute hypoxic respiratory failure.
Treatment Course – Opioid Use Disorder	Initially on oxycodone and hydromorphone. Transitioned to methadone, then cross-tapered to buprenorphine. Stopped buprenorphine and restarted methadone during a readmission.
Treatment Course – Malignancy	Began radiation and chemotherapy while inpatient. Stayed inpatient for the majority of her treatments. Imaging demonstrated marked disease improvement.

## Discussion

This was a case of significant medical complexity in the setting of OUD and lack of housing. In addition to her medical specialists, the patient was seen by a multidisciplinary team for these concerns, specifically, including Addiction Medicine, Palliative, and Psychiatry. She had multiple types of pain: (1) nociceptive (acute post-operative and cancer-related), (2) neuropathic (secondary to frostbite in all extremities), and (3) nociplastic (related to the recent death of her husband; adjusting to the new diagnoses of cancer, STEMI, stroke, and HFrEF; and placement of an ostomy). Management of these multiple pain syndromes in the setting of significant medical and social determinants of health-related complexity necessitated collegiality, collaboration, and advocacy on the patient’s behalf from all involved teams.

A large cross-sectional study evaluating the association between housing status and inpatient care delivery and outcomes among adults with cancer found that, despite higher rates of moderate or major illness severity and longer length of stay, people experiencing homelessness (PEH) were less likely to receive invasive procedures and systemic therapy [7]. However, PEH were more likely to receive radiotherapy while inpatients. Radiation therapy has been found to be effective in patients with gynecologic malignancies, even with baseline low performance status [8]. In our case, the patient benefited significantly from inpatient radiation therapy, as she was at high risk for loss to follow-up and cancer progression had she been discharged. Her multiple readmissions underscore the improbability of patients with high degrees of medical complexity being able to survive outside the hospital in unstable housing situations, let alone complete recommended outpatient follow-up and treatment regimens. While the barriers this patient encountered were particular to her case of cancer, pain, substance use, and homelessness, the challenges she faced in continuing care outside the hospital also exist for many PEH with moderate-to-severe chronic illness and mental health or substance use comorbidities.

Guidance for management of OUD in patients with advanced cancer is relatively nascent. Both buprenorphine and methadone have been recommended for treating cancer pain [9] and provided additional benefit in simultaneously treating this patient’s OUD. One qualitative study with palliative and addiction clinicians found consensus around the appropriateness of beginning treatment with buprenorphine/naloxone for patients with untreated OUD and cancer, regardless of prognosis; for those with shorter prognoses, beginning split-dose methadone was thought to be appropriate [10].

Generally, buprenorphine can be effective for treating cancer pain, especially in patients with renal impairment; however, its partial agonist property imparts a ceiling effect to analgesia [9]. Methadone, as a full agonist, does not share buprenorphine’s ceiling effect. At doses above 120 mg, it has concerns for QTc prolongation and torsades de pointes, and alternate opioids are considered recommended but not needed for those with a QTc of 450 to 500 msec [9]. Its use is also complicated by regulations requiring patients to visit Opioid Treatment Programs daily to receive their dose, which can be particularly difficult for PEH, patients with mobility challenges, and those without reliable transportation. However, as in this patient’s case, the choice of medication must be individualized for each patient and account for the patient’s entire clinical picture.

## Conclusions

The lessons learned include: (1) in patients with opioid use disorder and severe chronic cancer pain, shared decision-making with the patient and multi-specialty teams are vital to optimize patient safety and individualized health outcomes; (2) advanced multidisciplinary collaboration, care coordination, patient advocacy, and close follow-up are critical aspects of quality care for patients with significant medical complexity and social determinants of health barriers; and (3) adequate multi-system pain management and adaptable care plans can be effective tools for success among patients at high risk for loss to follow-up if discharged prematurely. It can be difficult to accommodate all of a patient’s care-related needs in existing post-discharge options.
